# Amplicon-based microbiome study highlights the loss of diversity and the establishment of a set of species in patients with dentin caries

**DOI:** 10.1371/journal.pone.0219714

**Published:** 2019-07-31

**Authors:** Diana Wolff, Cornelia Frese, Kyrill Schoilew, Alexander Dalpke, Bjoern Wolff, Sébastien Boutin

**Affiliations:** 1 Department of Conservative Dentistry, Centre of Dentistry, Oral Medicine and Maxillofacial Surgery, University of Tuebingen, Tuebingen, Germany; 2 Department of Conservative Dentistry, School of Dental Medicine, Ruprecht-Karls-University of Heidelberg, Heidelberg, Germany; 3 Medical Microbiology and Hygiene, Technical University Dresden, Dresden, Germany; 4 Department of Infectious Diseases-Medical Microbiology and Hygiene, University Hospital Heidelberg, Heidelberg, Germany; University of the Pacific, UNITED STATES

## Abstract

**Objectives:**

To elicit patterns in pathogenic biofilm composition we characterized the oral microbiome present in patients with dentin caries in comparison to healthy subjects.

**Methods:**

16S amplicon sequencing was used to analyse a total of 56 patients; 19 samples of carious dentin (pooled from at least three teeth) and 37 supragingival samples (pooled from three healthy tooth surfaces). Oral and periodontal status and socio-demographic parameters were recorded. Group assignment, smoking and further socio-demographic parameters were used as explanatory variables in the microbiome composition analysis.

**Results:**

Overall, a total of 4,110,020 DNA high-quality sequences were yielded. Using a threshold of similarity >97% for assigning operational taxonomic units (OTU), a total of 1,537 OTUs were identified. PERMANOVA showed significant differences in microbiome composition between the groups caries/healthy (p = 0.001), smoking/non-smoking (p = 0.007) and fluoride intake during childhood yes/no (tablets p = 0.003, salt p = 0.023). The healthy microbiome had a significantly higher diversity (alpha diversity, p<0.001) and a lower dominance (Berger-Parker index, p<0.001). It was dominated by *Fusobacteria*. A linear discriminant analysis effect size (LEfSe) yielded a set of 39 OTUs being more abundant in carious dentin samples, including *Atopobium* spp. *(14*.*9 log2FoldChange)*, *Lactobacillus casei (11*.*6)*, *Acinetobacter* spp. *(10*.*8)*, *Lactobacillus gasseri (10*.*6)*, *Parascardovia denticolens (10*.*5)*, *Olsenella profusa (10*.*4)*, and others. Also *Propionibacterium acidifaciens (7*.*2)* and *Streptococcus mutans (5*.*2)* were overabundant in caries lesions.

**Conclusions:**

The healthy microbiome was highly diverse. The advanced caries microbiome was dominated by a set of carious associated bacteria where *S*. *mutans* played only a minor role. Smoking and fluoride intake during childhood influenced the microbiome composition significantly.

**Clinical significance:**

The presented investigation adds knowledge to the still not fully comprehended patterns of oral microbiomes in caries compared with oral health. By analysing the genetics of biofilm samples from oral health and severe tooth decay we found distinct discriminating species which could be targets for future therapeutic approaches.

## Introduction

Microbiome analysis using Next Generation Sequencing (NGS) technology can unravel complex compositions of oral biofilms and so far helps finding non-culturable and “overlooked” bacteria [1–5]. Having insight into biofilm compositions at different sites and under various environmental or pathologic conditions is mandatory for further understanding of this extremely complex eco-system.

About 10 years ago, with the technological “OMICS”-breakthrough, Keijser *et al*. were the first to report on oral microbiome sequencing [6], subsequently presenting data on oral core microbiome composition [7]. Shortcomings of the first data sets were small sample size lacking representative character. The sequencing technologies then developed with impressive speed. Methodological shortcomings like DNA extraction and pre-amplification bias, sample contamination, and outcome effects by sequencing platforms and post-processing databases, are not yet solved, but improved. The enormous cost reduction made sequencing of large sample size possible.

For many decades, it was discussed whether mutans streptococci were the primary etiologic agent in cariology research, the “bad boys” of the caries process [8–10] or not. In recent years, molecular studies have unravelled that other genera, like *Lactobacilli*, *Actinomyces*, *Bifidobacteria*, *Veillonella*, *Cutibacteria* (formerly *Propionibacteria)* and *Atopobia* play significant roles in the pathology of dental caries, and hereby acting synergistically with or antagonistically against mutans streptococci [11–15]. Our research group previously detected *Propionibacterium acidifaciens* as being approximately 40-fold more abundant than *Streptococcus mutans* in deep caries samples [16]. Today, researchers widely agree that mutans streptococci serve as a good marker for disease, but not necessarily as the only and exclusive etiologic agent [17]. Also, the concept emerged that a healthy oral flora shows a rich and diverse homeostasis, which is narrowed down to a smaller range of a few outcompeting acidogenic and aciduric members in carious lesions [18].

Distinct patterns in healthy and diseased biofilm composition are yet to be determined. This complex research task is further challenged by the vast inter-individual differences in biofilm composition [19], which seem to get even more diffuse when biofilms enter a diseased stage. To gain a representative picture this study used comparably large cohorts of patients and evaluated the genetic composition of microbiome samples from healthy patients and patients with severely decayed teeth. Since caries is an ecologically driven disease [20], we recorded clinical and epidemiological parameters with a possible effect on microbiome composition, and used them for correlation analyses.

The hypothesis was that there would be differences in biofilm composition, diversity and dominance between healthy plaque and carious lesions.

## Material and methods

### Subject population

Adult subjects with severe dentin caries (n = 19) and caries-free controls (n = 37) were recruited consecutively from the Department of Conservative Dentistry at the University Clinic of Heidelberg between 2008 and 2016. Caries subjects required at least three dentin carious lesions and a DMFT>4. Caries-free subjects had to be free of caries for at least two years displaying a DMFT of 0–4. The recruited subjects were free of periodontal disease and any kind of systemic disease. The study protocol was approved by the Human Ethics Committee of the Medical Faculty of the University of Heidelberg (S-453/2007; S-079/2014). All subjects gave written informed consent. They underwent clinical examination to document the dental and periodontal status. Epidemiological data were taken from the patient records.

### Sampling

Healthy subjects had to abstain from oral hygiene, mouth rinsing and chewing gum consumption for at least 24 hours prior to sampling. Before sampling was started, cotton rolls were placed in the oral cavity to avoid saliva contamination. On tooth sites with visible plaque, supragingival samples were swiped off using a sterile periodontal probe or curette. This was done from at least four different tooth surfaces in different locations of the mouth. Patients with at least three radiographically detectable dentin carious lesions were scheduled for restorative treatment. During this treatment, the carious lesions were isolated using rubber dam and then excavated with a spoon excavator or with a round bur at slow speed. Supragingival plaque from healthy subjects and carious dentin samples from diseased subjects were pooled, respectively, in a sterile, 1.5-ml micro centrifuge tube and frozen at -20°C. To avoid DNA degradation the samples were further processed as quickly as possible within a maximum of 7 days.

### Isolation of bacterial DNA

Bacterial DNA was isolated following published protocols [21, 22] and by using a modification of the QIAamp DNA Mini Kit (Appendix D: protocols for bacteria; isolation of genomic DNA from gram-positive bacteria; Qiagen, Hilden, Germany).

### Library preparation for next generation sequencing (NGS)

DNA was amplified using universal bacterial primers targeting the V4 region of the 16S rRNA gene (515F and 806R from [23]). Each primer was barcoded to assign the sequences to the samples. PCR reaction mix contained Q5 High-Fidelity 1X Master Mix (New England BiolabsGmbH, Germany), 0.5 μM of each primer, 2 μL of DNA, and sterile water for a final volume of 25 μL. The thermal reaction was as follows: The first denaturation at 94°C for 3 min, followed by 30 amplification cycles (94°C for 45 sec, 50°C for 1 min, and 72°C for 1 min 30 sec), and the final extension at 72°C for 10 minutes (cycler: Primus 25, Peqlab Biotechnologie GmbH, Germany or FlexCycler^2^, Analytik Jena AG, Germany). Proper negative controls were processed in parallel to control contamination using sterile water as template. PCR products were evaluated by agarose gel electrophoresis (2%) for presence of amplicons and then purified by using Agencourt AMPure XP beads (Beckman Coulter, Germany) according to the manufacturer’s instructions. Purified products were checked for quality and concentration using the Quant-iT PicoGreen dsDNA Assay Kit (ThermoFisher Scientific GmbH, Dreieich, Germany) and the Bioanalyzer (Agilent Technologies Inc., Böblingen, Germany). Equimolar mix of all the PCR products was then sent to GATC Biotech (Konstanz, Germany), which performed the ligation of the sequencing adapters to the library and the paired-end sequencing on an Illumina Miseq sequencing system with 250 cycles.

### Analysis of sequences

Paired sequences were assembled with the following parameters: A minimum overlap of 100 nt and a maximum mismatch of 5 nt. Contigs were then filtered for quality; sequences with a quality score lower than 30 over 97% of the length were discarded. Each contig was assigned to the sample with the barcodes on both the right and left end (allowing no mismatch per barcode) using MOTHUR software [24]. Sequences were cleaned from ambiguity (no ambiguity allowed) and homopolymers (the maximum homopolymer length allowed: 8nt). Chimera detection was done by using the algorithm Uchime (Edgar *et al*. 2011). Clean sequences were subsampled to attain the same number of sequences for each sample (n = 4432), and Good’s estimator of coverage was calculated to ensure that coverage was sufficient. Sequences were clustered as Operational Taxonomic Units (OTU) (using a divergence threshold of 3%), and representative sequences were then classified at taxonomic levels by alignment with sequences from the Human Oral Microbiome Database (HOMD) (Bootstrap cut off at 80%).

### Statistical analysis

Various indices were calculated from the microbiota data: alpha diversity (non-parametric Shannon index), richness (Chao1 richness estimate, number of OTUs observed), evenness (non-parametric Shannon index-based measure of evenness) and dominance (Berger-Parker index). Beta diversity was assessed by calculating distance matrices based on Morisita-Horn distances and visualized by Principal Coordinates Analysis (PCoA). A PERMANOVA analysis was performed to assess the statistical significance of differences in explanatory variables among samples or groups of samples. All assessed clinical and epidemiological parameters, (Table 1) related to either caries or health, were considered in the PERMANOVA analysis. To detect differentially abundant OTUs between groups, linear discriminant analysis (LDA) effect size (LEfSe) analysis was performed. All statistical analyses were performed with MOTHUR 1.33.0 [24] and R 3.1.2 [25].

**Table 1:** Descriptive statistics on age and gender of patients.

		Subjects	Gender		Age (years)				
			Female	Male	Mean	Median	Standard deviation	Minimum	Maximum
Group	Overall	56	33	23	31.4	27.5	7.9	20.3	68.5
	Health	37	26	11	28.4	26.9	4.6	22.0	52.0
	Caries	19	7	12	37.2	32.7	11.9	20.3	68.5

## Results

**Fig 1:**
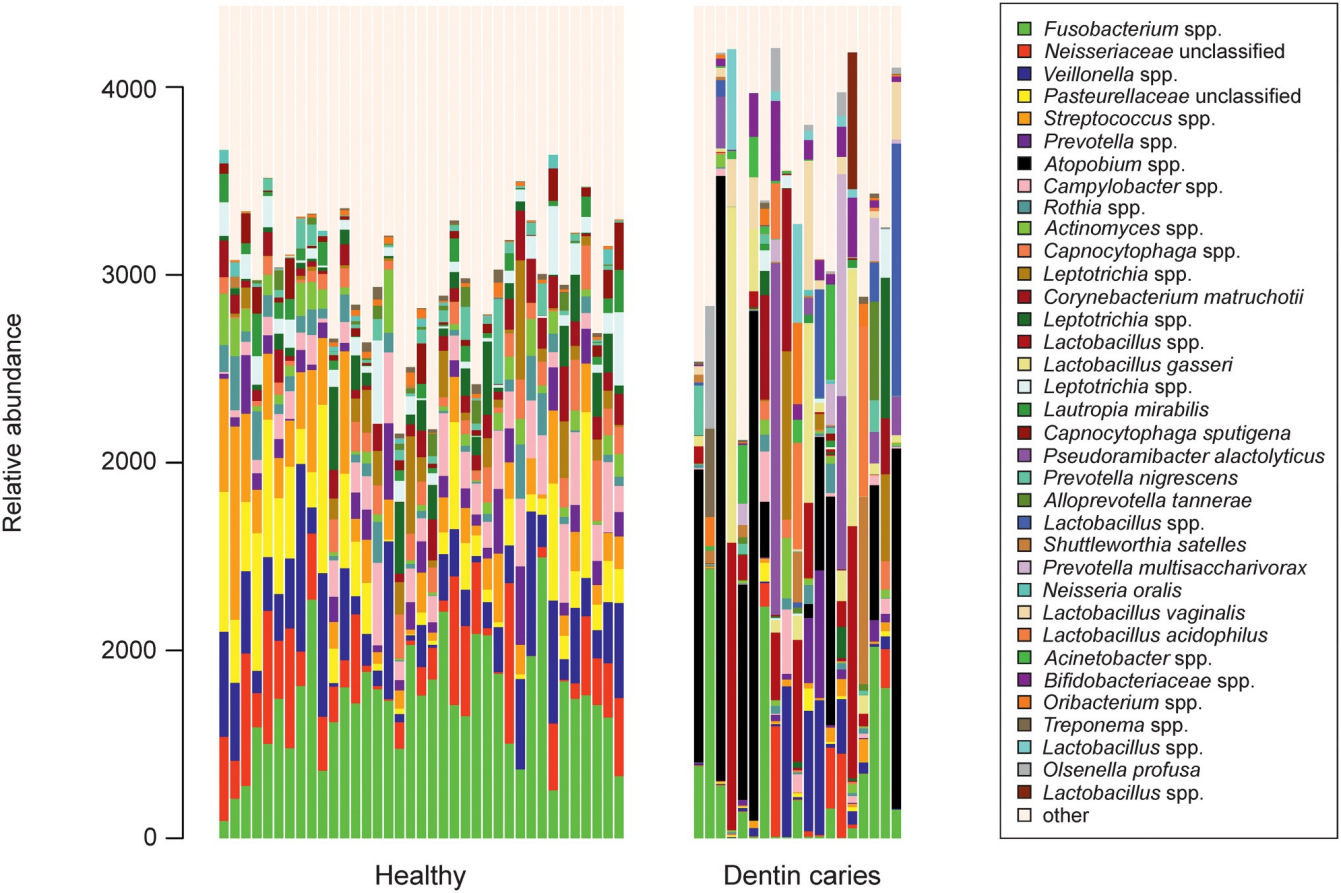
Microbiome structure. Microbiome structure of the 56 oral samples. Only the relative abundance of the 35 more abundant species are plotted, the rest are named “other”.

Thirty-three women and 23 men with a mean age of 31.4 years participated (for further parameters of the cohort see Table 1). Overall, 56 pooled oral samples were obtained from all participants yielding 4,110,020 DNA high-quality sequences. Using a distance-based similarity of >97% for operational taxonomic unit (OTU) assignment, a total of 1,537 OTUs were identified. The ten most abundant species detected among all samples were *Fusobacterium* spp., *Neisseriaceae unclassified*, *Veillonella* spp., *Pasteurellaceae unclassified*, *Streptococcus* spp., *Prevotella* spp., *Atopobium* spp., *Campylobacter* spp. *Rothia* spp., and *Actinomyces* spp. (Fig 1). The pattern of species distribution in healthy specimens seems well organized. In caries samples single and varying species dominate the individual samples in a seemingly random distribution (Fig 1).

**Fig 2:**
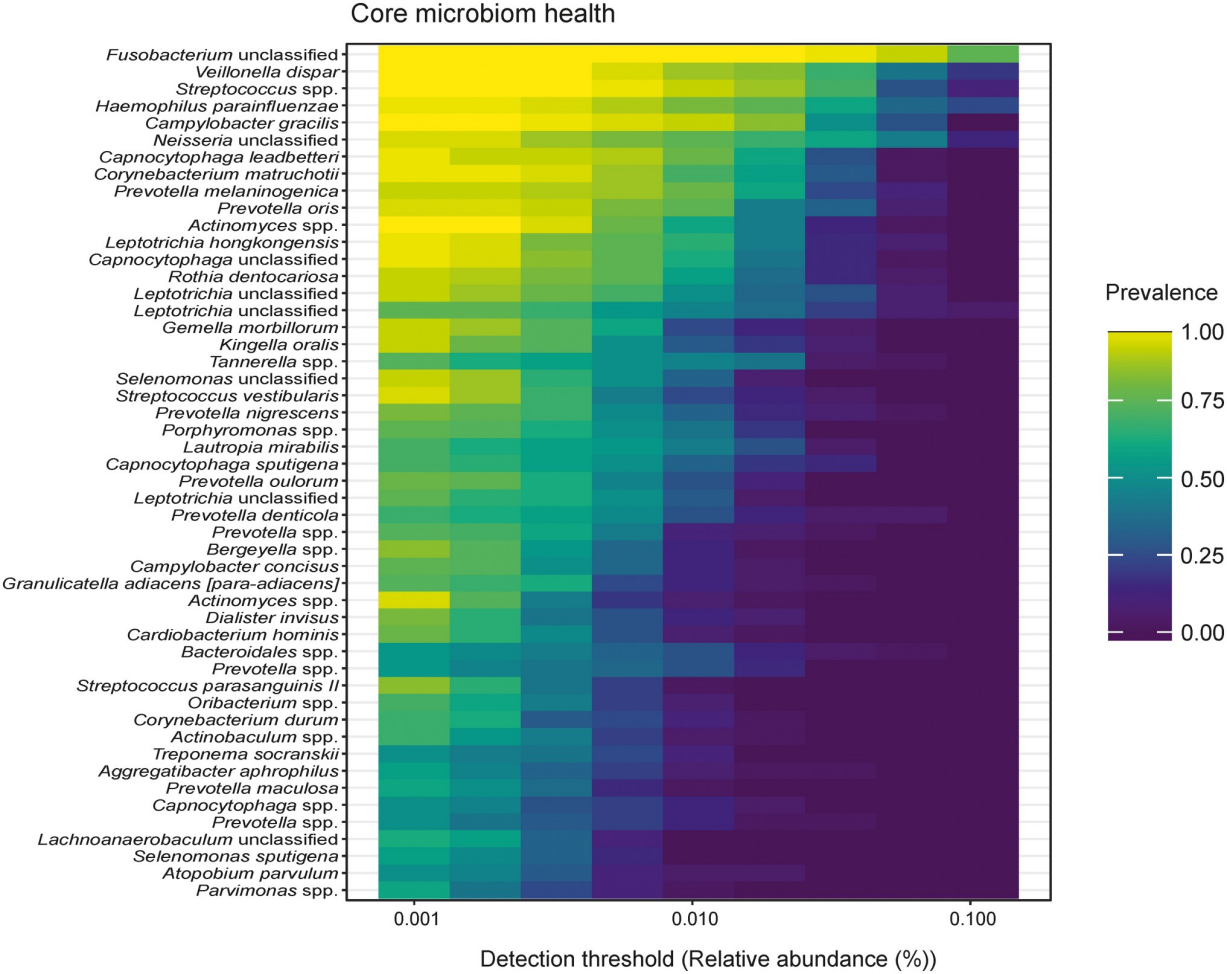
Heat map of the core microbiome in health. The healthy core microbiome is displayed by a heat map that identifies *Fusobacteria* unclassified, *Veillonella dispar*, *Streptococcus* spp., *Haemophilus parainfluenzae*, *Campylobacter gracilis*, *Neisseria* unclassified, *Capnocytophaga leadbetteri*, *Corynebacteriuim matruchotii*, *Prevotella melaninogenica*, and *Prevotella oris* as the ten most prevalent species.

A heatmap of the healthy core microbiome (Fig 2) displays *Fusobacteria unclassified*, *Veillonella dispar*, *Streptococcus* spp., *Haemophilus parainfluenzae*, *Campylobacter gracilis*, *Neisseria unclassified*, *Capnocytophaga leadbetteri*, *Corynebacteriuim matruchotii*, *Prevotella melaninogenica*, and *Prevotella oris* as the ten most prevalent.

**Fig 3:**
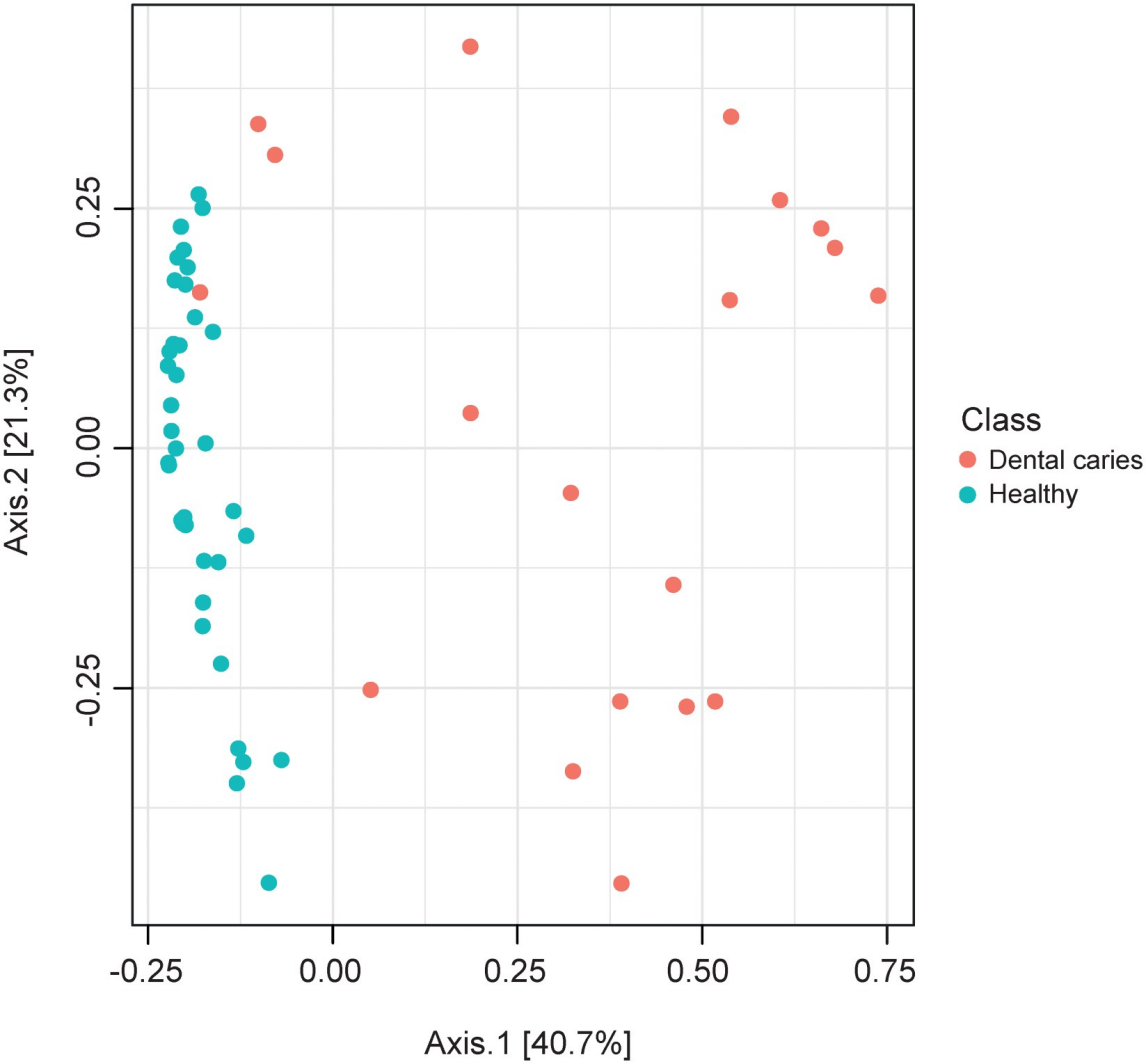
Similarity of microbiome composition. Scatter plot of the Principal Coordinates Analysis (PCoA) using the Morisita-Horn dissimilarity index.

**Fig 4:**
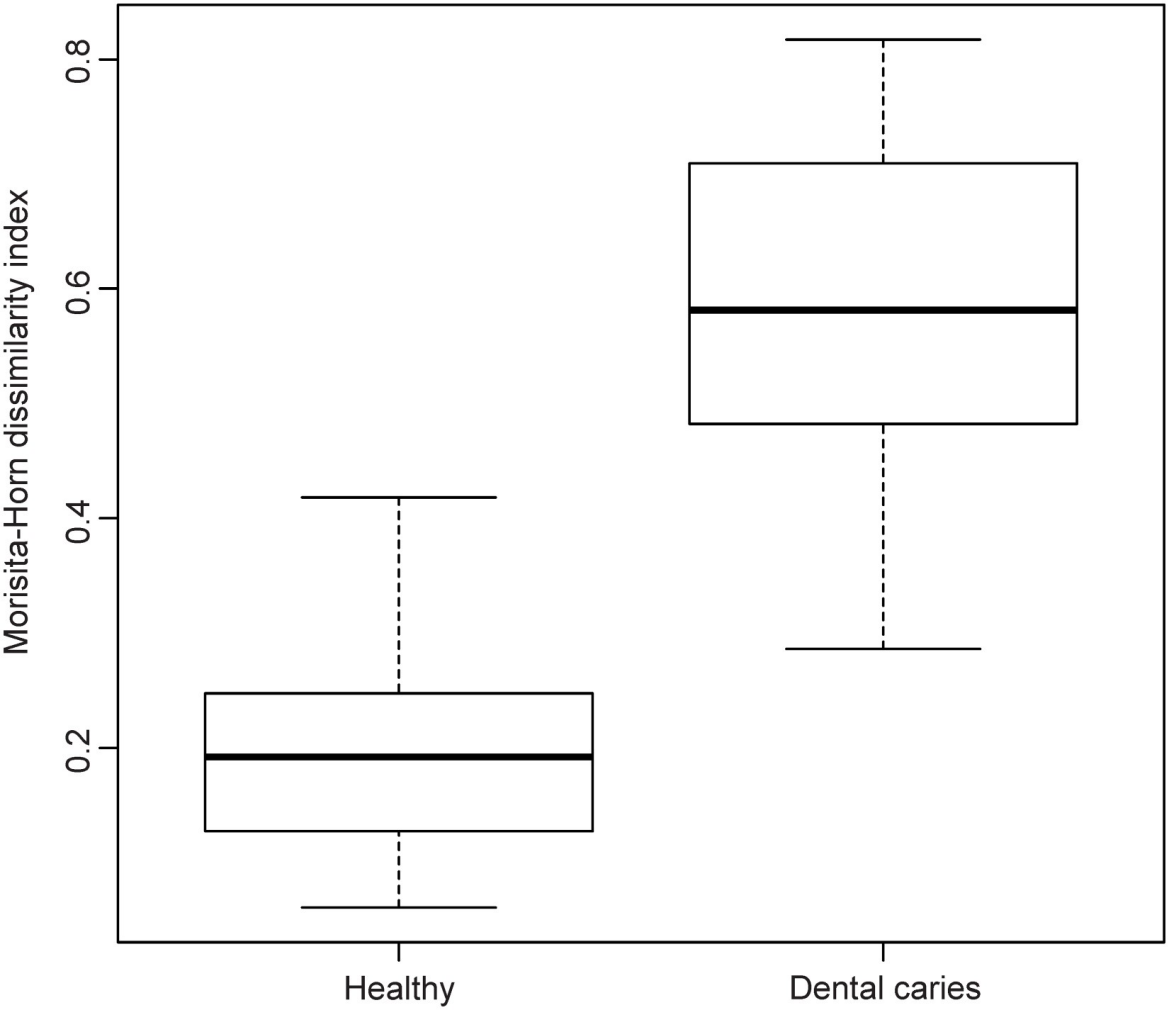
Similarity of microbiome composition. Boxplots of Morisita-Horn distances.

The similarity between microbiome structure of the specimens was studied by Principal Coordinates Analysis (PCoA) using the Morisita-Horn dissimilarity index. In the PCoA plot the healthy samples clustered homogenously due to higher composition similarity, whereas caries specimens scattered diffusely (Figs 3 and 4).

**Fig 5:**
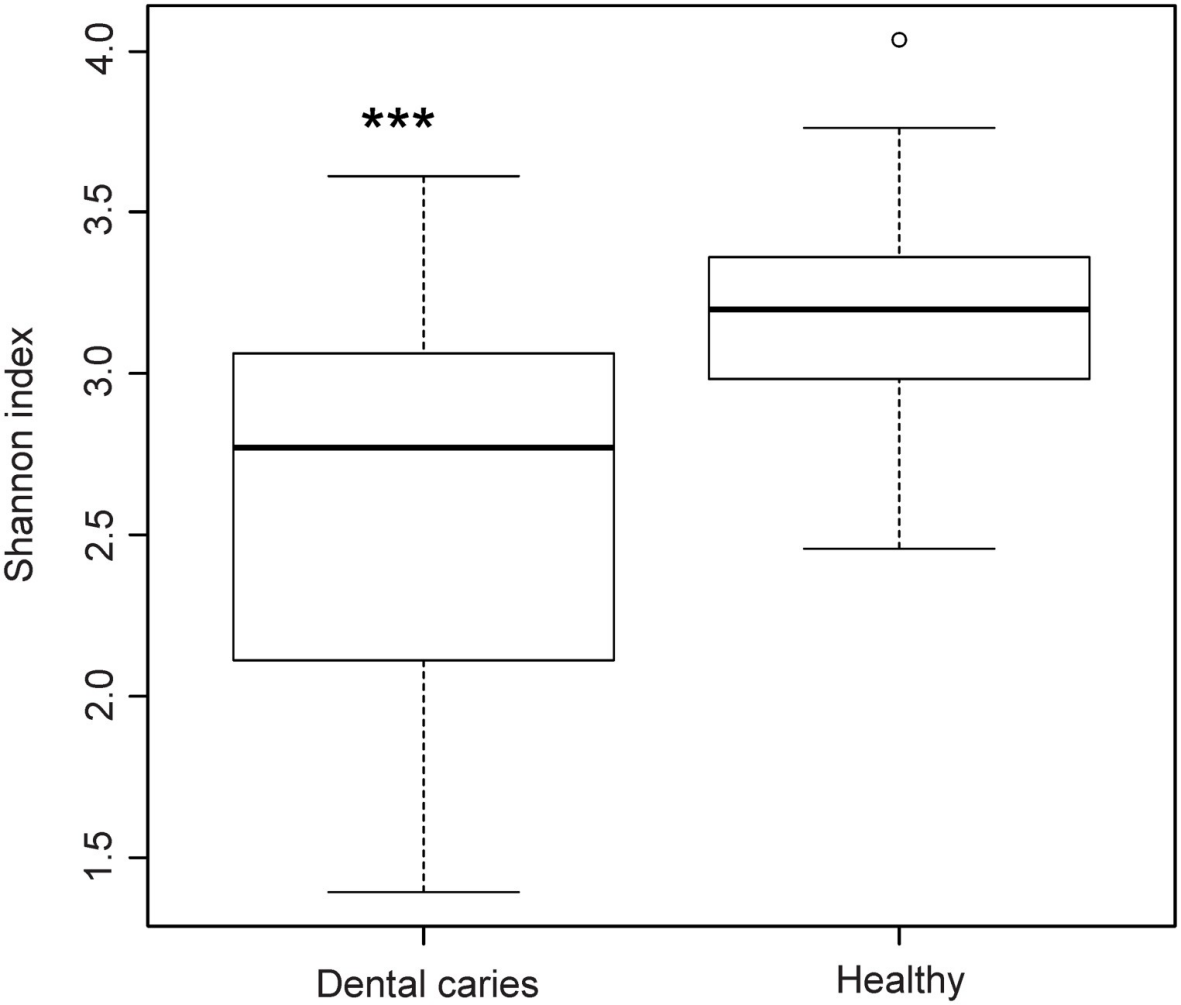
Microbial diversity. Boxplot of the non-parametric Shannon index evaluating the effect of group assignment on microbial diversity.

**Fig 6:**
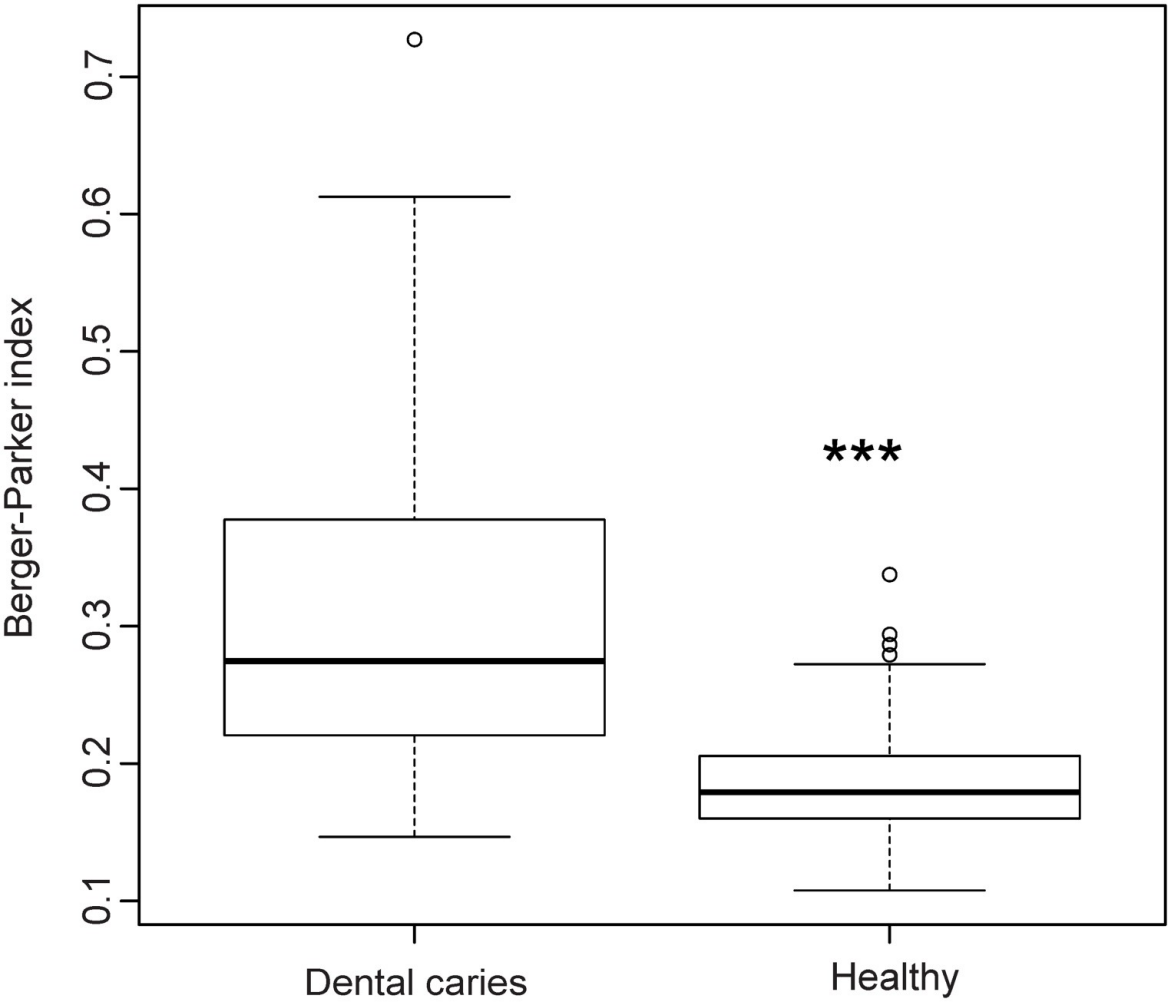
Microbial dominance. Boxplot of the Berger-Parker index evaluating the effect of group assignment on microbial dominance.

We then evaluated the effect of group assignment (caries vs. healthy) on microbial diversity (Non-parametric Shannon index) and on microbial dominance (Berger-Parker index). The Shannon index, which reflects a more diverse microbiota with increasing values, was significantly higher for the healthy samples. Berger-Parker evaluates the relative abundance of the dominant OTU. It can, therefore, be interpreted as a marker for biofilm dysbiosis allegorizing single species dominance in the environment. Our data displayed a highly significant difference in alpha diversity between the groups (p<0.001) and a significantly higher dominance in the diseased samples versus healthy samples (p<0.001) (Figs 5 and 6).

**Fig 7:**
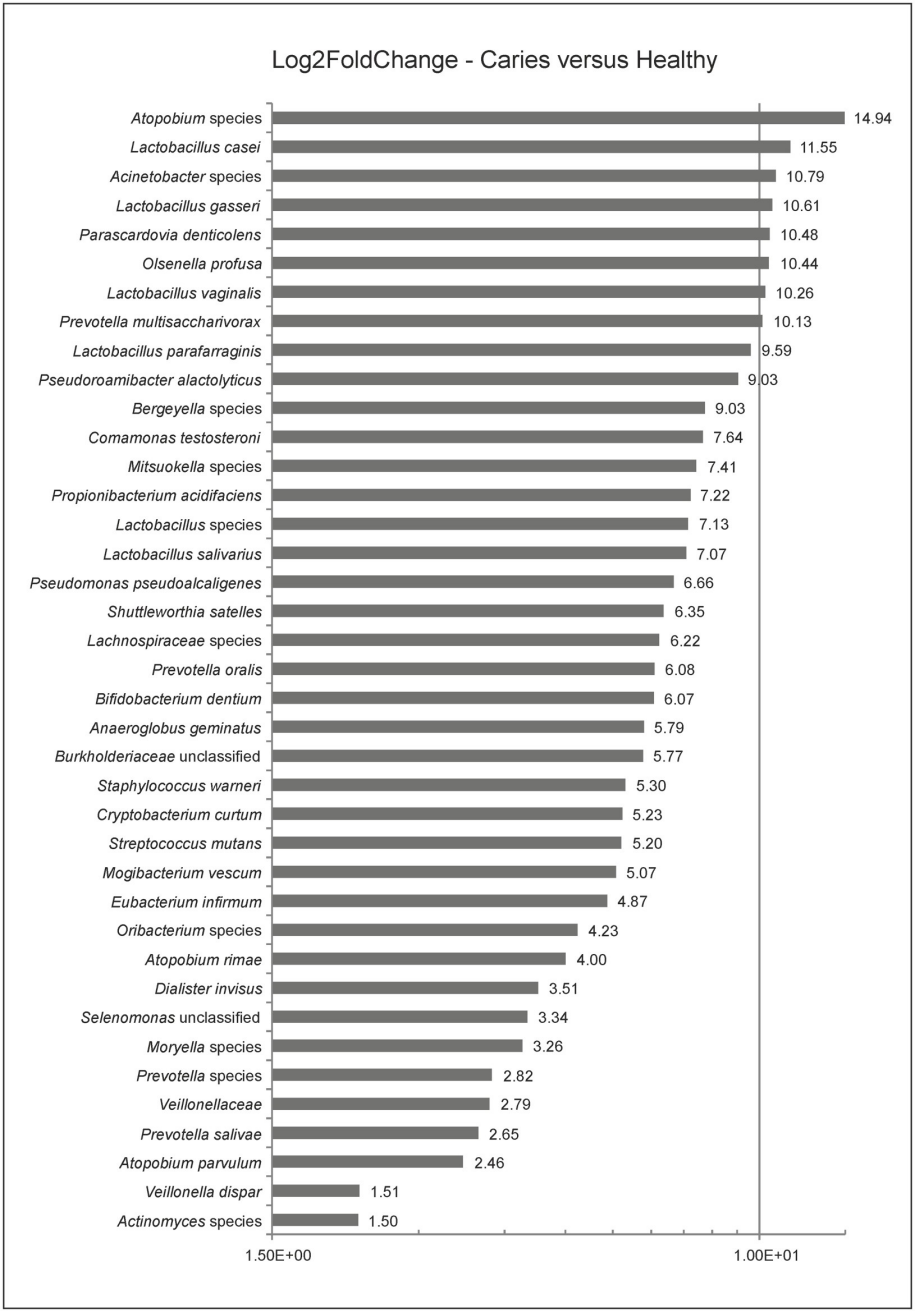
Differences in microbiome composition. Log2FoldChange bar charts of linear discriminant analysis effect size (LEfSe) using the distinctive parameter “class” to test for OUT differences.

To screen for differences in microbiome compositions between the two groups, a linear discriminant analysis effect size (LEfSe) was used to test for differences in the OTUs’ abundances. LEfSe yielded a set of 39 species being significantly more abundant in caries specimens (Fig 7). The log2 fold change ratios of these overabundant species varied between 1.5 and 14.94 (p<0.001 for all representatives). The top overabundant members of the investigated caries community were *Atopobium* spp. (14.94), *Lactobacillus casei* (11.55), *Acinetobacter* spp. (10.79), *Lactobacillus gasseri* (10.61), *Parascardovia denticolens* (10.48), *Olsenella profusa* (10.44), *Lactobacillus vaginalis* (10.26), *and Prevotella multisaccharivorax* (10.13). *Propionibacterium acidifaciens* (7.22) and *Streptococcus mutans* (5.2) ranged in the middle segment of the fold change range (Fig 7).

Analyses of the influence clinical and socio-demographic parameters on the microbial structure were done by non-parametric multivariate statistical testing (PERMANOVA). Significant values were yielded for class (caries vs. healthy; p = 0.001), smoking (yes/no; p = 0.007), DMFT (p = 0.001), Dt (p = 0.001), Mt (p = 0.002), Ft (p = 0.001), and fluoride tablet and salt intake during childhood (p = 0.003, p = 0.023, respectively). The parameter “fluoride” represented the present use of fluoridated oral hygiene products. With p = 0.066 it was, compared with the other parameters, the one with the highest tendency towards significance (Table 2).

**Table 2:** Descriptive statistics.

Factor	Df	SumsOfSqs	MeanSqs	F.Model	R2 (% of Variation Distance)	Pr(>F)	Code
Class (caries/healthy)	1	3.71	3.71	23.35	0.30	0.001	***
Smoker (yes/no)	1	0.74	0.74	3.48	0.06	0.007	**
Caries experience of parents (yes/no)	1	0.34	0.34	1.54	0.03	0.146	
Caries experience of siblings (yes/no)	1	0.42	0.42	1.93	0.03	0.111	
Dental visits per year	1	0.14	0.14	0.64	0.01	0.649	
DMFT	1	3.20	3.20	19.05	0.26	0.001	***
Dt	1	1.50	1.50	7.48	0.12	0.001	***
Mt	1	1.78	1.78	9.14	0.14	0.002	**
Ft	1	2.63	2.63	14.71	0.21	0.001	***
Fluoride tablets childhood (yes/no)	1	0.91	0.91	4.34	0.07	0.003	**
Fluoride salt childhood (yes/no)	1	0.65	0.65	3.00	0.05	0.023	*
Dental hygiene (times/day)	1	0.19	0.19	0.83	0.02	0.482	
Dental hygiene (minutes/tooth brushing)	1	0.25	0.25	1.10	0.02	0.353	
Chlorhexidine use (yes/no)	1	0.22	0.22	0.99	0.02	0.339	
Fluoride use (yes/no)	1	0.48	0.48	2.18	0.04	0.066	.
Tea consumption (yes/no)	1	0.36	0.36	1.62	0.03	0.163	
Sugar intake (times/day)	1	0.29	0.29	1.29	0.02	0.263	
Whole food (yes/no)	1	0.25	0.25	1.13	0.02	0.325	
Vegetarian (yes/no)	1	0.17	0.17	0.74	0.01	0.562	
Use of chewing gum (yes/no)	1	0.13	0.13	0.58	0.01	0.713	
Use of xylitol (yes/no)	1	0.06	0.06	0.26	0.00	0.937	
Use of calcium containing products (yes/no)	1	0.16	0.16	0.70	0.01	0.630	
Use of probiotics (yes/no)	1	0.25	0.25	1.13	0.02	0.324	

Descriptive statistics on class, dental status and epidemiologic parameters; epidemiological parameters were gained by questionnaires; clinical data was extracted from records. Codes *, **, *** represent p≤0.05, 0.05>p<0.001, and p≤0.001, respectively.

LEfSe was also used to test for OTU differences between smokers and non-smokers yielding a set of four species being more abundant in smokers. *Neisseria unclassified* (Log2 fold change 4.26), *Haemophilus parainfluenzae* (2.57), *Lautropia mirabilis* (3.66), and *Corynebacterium durum* (3.38) were overabundant in smokers (p-values <0.000, for all). A correlation network analysis yielded strong correlation between a set of species and fluoride intake during childhood (Table 3). Correlation coefficients (Rho) ranged from 0.42 to 0.54 for the intake of fluoride salt and from 0.42 to 0.6 for the intake of fluoride tablets in childhood. A correlation of 40–60% (Rho 0.4–0.6) is considered to be strong.

**Table 3:** Correlation network analysis.

Factor	Species	Rho	p-value
Fluoride salt intake during childhood	Haemophilus parainfluenzae	0.42	0.001
	Streptococcus spp.	0.54	0.000
	Rothia dentocariosa	0.46	0.000
	Gemella morbillorum	0.44	0.001
	Granulicatella adiacens	0.44	0.001
	Actinobaculum spp.	0.46	0.000
Fluoride tablet intake during childhood	Haemophilus parainfluenzae	0.60	0.000
	Gemella morbillorum	0.43	0.001
	Alloprevotella spp.	0.50	0.000
	Bergeyella spp.	0.42	0.001
	Abiotrophia defectiva	0.46	0.000

Results of the correlation network analysis yielded a correlation between two parameters and a set of species.

## Discussion

The presented investigation adds data to the still not comprehended patterns of oral microbiome composition in caries compared with oral health [26]. By analysing biofilm samples from patients with oral health and patients with severe tooth decay we aimed at further describing “Who is there?” Furthermore, it was our interest to investigate how the clinical and epidemiological parameters relate to microbiome composition.

We found that the healthy microbiome displayed stable and repetitive character compared with the diseased ones. The healthy core community was dominated by *Fusobacteria*, *Veillonella*, *Streptococcus* spp., *Haemophilus parainfluenzae*, *Campylobacter gracilis*, *Neisseria unclassified*, *Capnocytophaga leadbetteri*, *Corynebacteriuim matruchotii*, *Prevotella melaninogenica*, *Prevotella oris*, and thus confirms literature data [7]. These bacteria, and *Actinomyces* as well were already shown to be relatively constant and abundant within and between individuals [27]. In this study, the health associated core microbiome was higher in alpha diversity. Conflicting data exists on this parameter in concordant studies [28–30], but also in deviant ones [31–33]. Clearly, species richness is high in a healthy oral environment and narrows down to few out-competing pathogens in the course of a disease. In caries, the degree of dominance of pathogens, however, does not only depend on the extent of the disease but also on environmental factors during disease progression. For example, Johannsson *et al*. found that absence of dental care in a Romanian cohort led to an overabundance of *Streptococcus mutans* in caries lesions when compared to a Swedish cohort with access to preventive measures [32]. Fluoride application and professional dental care can successfully target mutans streptococci, yet disease still occurs. Substituting pathogens emerge and benefit from this effect. The resulting dysbiotic microbiomes are not as uniformly dominated by traditional pathogens, but show higher pathogenic diversity [34].

In the present investigation, deep dentin lesions showed higher dominance, less diversity (Figs 3 and 4), and a set of carious associated bacteria where *Streptococcus mutans* played a role of only minor importance (Fig 7). Eriksson *et al*. have tried to classify *Streptococcus mutans* with the presence of accompanying bacterial species in a kind of *Streptococcus mutans* abundance model [35]. Low *Streptococcus mutans* was associated with the presence of *Propionibacterium propionicum* [35]. *Propionibacteria* are found at many sites of the human body, also in the mouth. There they seem to be associated with dentin caries, especially *Propionibacterium propionicum* [35] and *Propionibacterium acidifaciens* [16]. This seems plausible since *Propionibacteria* are Gram-positive, anaerobic and generally producing lactic acid, propionic acid, and acetic acid from glucose. The fact that they are proteolytic and co-aggregating with Lactobacillus species gives them a competitive advantage in deep lesions where the glucose supply is limited and proteins are more frequently available by collagen degradation. In the present data set *Streptococcus mutans* is only moderately overabundant whereas *Propionibacterium acidifaciens* and diverse *Lactobacillus species* are the dominating species. Referring to the model of Eriksson *et al*., we seem to have the predominantly low *Streptococcus mutans*–high *Propionibacterium* (and *Lactobacillus*) pattern. Obata *et al*. also found that in their caries samples of 32 Japanese patients aged 4–76 years more than half of the mean bacterial distributions of bacterial genera in carious dentin were *Lactobacillus* and *Propionibacterium* [36]. What was also notable in this study was that the main species of the genus *Propionibacterium* was *Propionibacterium acidifaciens*. The data was analysed regarding high, middle and low Lactobacillus abundance clusters. The bacterial composition pattern in the cluster with middle *Lactobacillus* abundance was composed of high proportions of *Propionibacterium* and *Olsenella* pointing to a possible co-aggregation [36]. By now, evidence is increasing that *Streptococcus mutans* is an effective pathogen and highly prevalent in initial lesions. However, with progression of disease, *Streptococcus mutans* cannot persist against the “truly acid-tolerant heavyweights” (e.g., certain *Lactobacilli* and others [37], which drive the pH to such low values that no longer can be tolerated by less aciduric isolates of mutans streptococci [26]. Similar to the healthy oral core microbiome, the mystery of a microbiome for late stage diseases is disclosed more and more.

The correlation network analysis of the present study evaluated a strong correlation between fluoride intake during childhood and specific biofilm species (*Haemophilus parainfluenzae*, *Streptococcus* spp., *Rothia dentocariosa*, *Gemella morbillorum*, *Granulicatella adiacens*, *Alloprevotella* spp., *Bergeyella* spp., *and Abiotrophia defektiva*) (Table 3). The PERMANOVA analysis yielded those parameters as significant in affecting the microbiome composition, whereas the present fluoride intake by oral hygiene products was not (Table 2). When looking closer at this seemingly disconcerting result, an explanatory approach could be the fact that developed and individually stable adult microbiomes can be resilient [38], also against effects of antimicrobial substances [39–41]. However, there is a proven cariostatic effect of topically applied fluorides. A recent *in vitro* study emphasized that this effect did not manifest via biofilm modification or growth, but via mechanisms of acid production inhibition, EPS (extracellular polysaccharides) volume decrease, and/or via a shift in the de/remineralization balance [42]. Interestingly, our data points at a possible biofilm modifying effect of fluorides, when taken in form of tablets and salt during childhood biofilm development; an effect, which might last throughout a person’s lifetime. So far there is no study known to the authors that would have investigated this hypothesis, and data interpretation must be done very cautiously. Further research and discussion on this hypothesis is clearly necessary.

Under critical review, the study has some limitations. First, the sample size of the carious group is smaller than that of the healthy group. Furthermore, we compared two different sample materials, plaque and carious dentin. However, the study concept was a cross-sectional juxta positioning of the most differing states (oral health vs. late stage of carious disease) in order to find the main discriminating patterns. Also, it could be criticized that we focused on mere genetic information with no data on the vitality and metabolism of the species detected. However, we see strengths in the very strictly standardized clinical sampling protocol with high effort to avoid contamination, in the use of evidence based processing protocols, and in the assessment of a wide range of clinical and epidemiological data from the evaluated cohort. The latter helped to integrate the microbiome data in a reasonable co-variable context.

## Conclusion

The healthy oral core microbiome had a high alpha diversity, was stable and showed repetitive patterns of health representatives. *Fusobacteria* were the predominant species. Higher dominance and less diversity was seen in samples taken from individuals with advanced dentin caries, when compared with the oral microbiomes of caries-free individuals. Also, a set of caries associated bacteria was present. *Lactobacilli* were distinctly overabundant, *Streptococcus mutans* played a minor role. There might be an effect of fluoride intake during childhood on microbiome composition throughout life.
